# Complexity Reduction in Analyzing Independence between Statistical Randomness Tests Using Mutual Information

**DOI:** 10.3390/e25111545

**Published:** 2023-11-15

**Authors:** Jorge Augusto Karell-Albo, Carlos Miguel Legón-Pérez, Raisa Socorro-Llanes, Omar Rojas, Guillermo Sosa-Gómez

**Affiliations:** 1 Instituto de Criptografía, Facultad de Matemática y Computación, Universidad de la Habana, Habana 10400, Cuba; jorgekarellalbo@gmail.com (J.A.K.-A.); clegon58@gmail.com (C.M.L.-P.); 2 Facultad de Ingeniería Informática, Universidad Tecnológica de la Habana José Antonio Echeverría (CUJAE), Habana 19390, Cuba; raisa@ceis.cujae.edu.cu; 3 Facultad de Ciencias Económicas y Empresariales, Universidad Panamericana, Álvaro del Portillo 49, Zapopan 45010, Jalisco, Mexico; orojas@up.edu.mx

**Keywords:** mutual information, complexity, PRNG, cryptography

## Abstract

The advantages of using mutual information to evaluate the correlation between randomness tests have recently been demonstrated. However, it has been pointed out that the high complexity of this method limits its application in batteries with a greater number of tests. The main objective of this work is to reduce the complexity of the method based on mutual information for analyzing the independence between the statistical tests of randomness. The achieved complexity reduction is estimated theoretically and verified experimentally. A variant of the original method is proposed by modifying the step in which the significant values of the mutual information are determined. The correlation between the NIST battery tests was studied, and it was concluded that the modifications to the method do not significantly affect the ability to detect correlations. Due to the efficiency of the newly proposed method, its use is recommended to analyze other batteries of tests.

## 1. Introduction

In cryptographic applications, sequences of random numbers are very important. One of the widest-spread applications is the generation of secret keys. These sequences are obtained using random number generators, which, depending on the source of randomness, can be classified as pseudo-random number generators (PRNGs) or truly random number generators (TRNGs). Some authors, such as [1], include a third group of generators called hybrid random number generators (HRNGs). The HRNG combines elements of the previous two generators.

According to [2], the best way to generate “unpredictable” random numbers is to use physical processes, such as atmospheric or thermal noise, cosmic radiation, and some other phenomena; however, number generation from physical processes is relatively inefficient since TRNGs are expensive devices both in terms of execution and applicability. For this reason, ref [3] states that most systems use PRNGs instead of TRNGs.

Weak RNGs have been identified as the cause of many security problems in computer systems. A concrete case is exemplified by MIFARE Classic, an RFID radio frequency identification) card manufactured by NXP Semiconductors that had a weak PRNG, making it possible to attack their channel of supposedly secure communications [4]. Furthermore, ref [5] described pre-play and man-in-the-middle attacks that demonstrated how, due to a weak RNG, a payment system could be abused. An explanation of how it was possible to attack Taiwan’s national database of “Citizen Digital Certificates” was presented in [6], where it was due to poor implementation of the TRNG in the Office-certified Renesas AE45C1 v.01 smart card IC German Federal Information Security (BSI) [7]. These examples highlight the need for further analysis of RNG design and implementation to mitigate the possibility of such attacks in the future.

Because PRNGs are based on deterministic algorithms, it is necessary to examine the output to confirm its suitability for cryptographic applications. This output is statistically analyzed by one or several randomness tests, and the results are evaluated to determine whether the generator is random or not. The statistic associated with each statistical test attempts to identify the presence or absence of a specific pattern. If the pattern is detected, then the lack of randomness in the sequence can be inferred. There are many statistical tests for randomness. The same test can even be applied multiple times with different combinations of parameters [8].

Although some authors [9] recommend using multiple statistical randomness tests to leverage each test’s strengths, applying too many tests may lead to overestimating the properties of a PRNG. Thus, ref [10] suggest that two conceptually different tests may evaluate the same characteristic of randomness and produce correlated results. In addition, not only can the correlation between different randomness tests be studied, but the same test with different parameters can be correlated. Therefore, correlation analysis between randomness tests can also be used for parameter selection. In [11,12], the study of correlations between tests using the Pearson correlation coefficient (CCP) was proposed; however, this coefficient is not capable of detecting nonlinear correlations.

Results reported by [13] demonstrate the need for a greater variety of statistical tests. In their work, they studied eight RNGs, and, in some cases, generators showed difficulties in passing the tests of Alphabits, Rabbit, Small Crush, and Crush batteries, passing the tests of Dieharder and SP800-22. As [13] points out, future batteries of statistical tests should be thoroughly analyzed for possible correlations between their tests before publication. Likewise, a continuous review of the new randomness tests must be carried out to ensure their efficiency and statistical strength and integrate them into the batteries if they are not correlated with the existing tests or are superior to them.

In [12,14], Pearson’s correlation coefficient was used to analyze correlations between tests; however, [2,11] focused on the proportion of regions where *p*-values are less than 0.01. Then, ref [15] analyzed the dependencies between nine NIST tests and found the same dependencies as [12]. Another approach proposed by [16] analyzed the difference between *p*-values corresponding to two different tests and detected correlations according to the distribution of that difference.

In [14], dependencies between some NIST tests were determined using the Pearson correlation coefficient, and patterns were found in the evolution of these dependencies according to specific factors, such as the length of the sequences analyzed by the tests. In [17], a novel method was proposed to detect the correlation between statistical randomness tests using mutual information for the first time. This measure was used to examine the tests present in the NIST SP 800-22 battery, detecting new correlations.

Different articles have verified the effectiveness of mutual information in studying the correlation between the randomness tests of different batteries. In [18], DieHarder, TufTest, and SmallCrush batteries were studied, and in [19], the FIPS 140-2 battery was analyzed using the method proposed in [17]. Mutual information can detect the correlations found by Pearson correlation and new nonlinear dependencies between tests. In [20], the complexity of the method proposed in [17] was estimated, and its high complexity was pointed out as a disadvantage, which prevents testing batteries with more tests or longer sequences. However, mutual information as a new measure of independence between randomness tests represents a significant advance and an important piece for building a definitive test battery [18].

The main objective of this work is to reduce the complexity of the method proposed by [17], which we will call from now on MIRT-1 (Mutual Information to analyze Statistical Randomness Test v1). The step in which the mutual information values corresponding to significant correlations are determined is modified to achieve this. The complexity reduction achieved with this modification is estimated theoretically and then confirmed experimentally. The efficiency of the new proposed method (MIRT-2) will be determined by considering the complexity calculation. On the other hand, the efficacy will be measured according to the number of correlations that it can detect compared to the MIRT-1 method.

## 2. Preliminaries

### 2.1. Mutual Information

Mutual information (MI) is an important measure of statistical correlation. It stands out for its ability to detect the correlation between variables and possesses properties that make it an ideal measure of stochastic dependence [21,22,23,24]. Unlike the Pearson correlation coefficient (PCC), which only considers linear dependencies, or other correlation coefficients that only detect monotonic dependencies, MI considers all correlations regardless of their functional form.

The MI between two discrete random variables *X* and *Y* is defined as

(1) $$MI\left(X,Y\right)=\sum_{y\in Y}\sum_{x\in X}p\left(x,y\right)\log\left(\frac{p(x,y)}{p(x)p(y)}\right),$$

where p(x,y) is the joint probability function of *X* and *Y*, and p(x) and p(y) are the marginal probability distribution functions of *X* and *Y*, respectively. MI can also be defined in terms of entropy as

(2) MI(X,Y)=H(X)+H(Y)−H(X,Y),

where H(X) and H(Y) are the marginal entropy of the variables *X* and *Y*, respectively, and H(X,Y) is the joint entropy of both variables. For the case of continuous random variables, the sums are replaced by integrals:

(3) $$MI\left(X,Y\right)=\int_{Y}\int_{X}p\left(x,y\right)\log\left(\frac{p(x,y)}{p(x)p(y)}\right)\;dxdy.$$

More on the properties of entropy and mutual information for continuous variables and their relationship to the discrete case can be found in [25].

### 2.2. Estimating Mutual Information

When the probability distributions are unknown, it is not possible to calculate the exact value of MI(X,Y), so it is necessary to calculate a sample estimator $\hat{MI}\left(X,Y\right)$. Estimators of the mutual information $\hat{MI}$ differ in estimating the probabilities of the marginal and joint densities. Some of the estimators proposed in the literature use discretization [26,27], kernels [28,29] or correlation integrals [30], k-nearest neighbors [31,32], B-splines [33], or Gram–Charlier polynomial expansion [34].

One of the simplest estimators is the maximum-likelihood (ML) estimator (plug-in). With this, entropy is estimated from the observed individual and joint frequencies. Since there are no assumptions about the data distribution, the ML estimator is considered non-parametric. Another of the estimators used is the James–Stein shrinkage estimator. The approach of this estimator is considered semi-parametric because it has characteristics of parametric and non-parametric methods [35]. A comprehensive comparison of estimators can be found in [35,36].

According to [37], the most common mutual information estimator is the naive equidistant binning estimator. This estimator considers each variable’s domain partition in a finite number, *n*, of discrete intervals (equidistant partition). The number of intervals for each variable is the same, so the parameter to optimize is the number of intervals to discretize or, equivalently, the length of the interval. The binning process begins with selecting the support interval, (a,b). Usually, this interval is constructed from the smallest and largest values in the sample $\left\{x_{1},x_{2},\ldots ,x_{n}\right\}$. Given the interval (a,b), a frequency histogram is constructed from the number of points in each interval. The intervals, $B_{k}$, are half-open, i.e., $B_{k}=\left[t_{k},t_{k+1}\right)$ for k=−∞,…,−1,0,1,…,∞, where the points $t_{k}$ satisfy that $t_{k}<t_{k+1}$. The width of each interval is denoted by $h_{k}=t_{k+1}-t_{k}$, and the number of elements in the interval is denoted by $v_{k}$. In this way, given the sample $x_{1},x_{2},\ldots ,x_{n}$, we have

(4) $$v_{k}=\sum_{i=1}^{n}1_{x_{i}\in B_{k}},$$

where 1(·) is the indicator function. It is clear that $v_{k}\geq 0$ and $\sum_{k}v_{k}=n$. Generally, the intervals are selected so they are of the same width, i.e., $h_{k}=h,\;\forall k$. For equal-width intervals, we define

(5) $$h=\frac{b-a}{k}.$$

Many researchers have attempted to determine the optimal value, *k*, of the intervals, but these methods often rely on strong assumptions about the distribution of the data [38]. Depending on the distribution type and the analysis objective, different values of *h* can be selected. Experimentation is usually necessary to determine the optimal width. Table 1 presents some of the most used rules for selecting *k*.

In most cases, the number of bins to discretize is set to k=10 [50]. This work will discretize it in k=10 intervals because it is the same approach used in [17] to design the MIRT-1 method.

### 2.3. Distribution of Estimators

The ML estimator of mutual information ${\hat{MI}}_{ML}\left(X,Y\right)=$${\hat{H}}_{ML}\left(X\right)+{\hat{H}}_{ML}\left(Y\right)-{\hat{H}}_{ML}\left(X,Y\right)$ has been extensively studied in the literature [51,52]. It is known from [51] that, under certain conditions, such as finite alphabet size and MI>0, the following holds:

(6) $$\frac{\sqrt{n}\left({\hat{MI}}_{ML}-MI\right)}{\hat{\sigma }}∼N\left(0,1\right).$$

On the other hand, in [52,53], it was stated that

(7) $$2n{\hat{MI}}_{ML}∼\chi_{\left(I-1)(J-1\right)}^{2}$$

where *I* and *J* are the sizes of the alphabets of *X* and *Y*, respectively.

**Figure 1:**
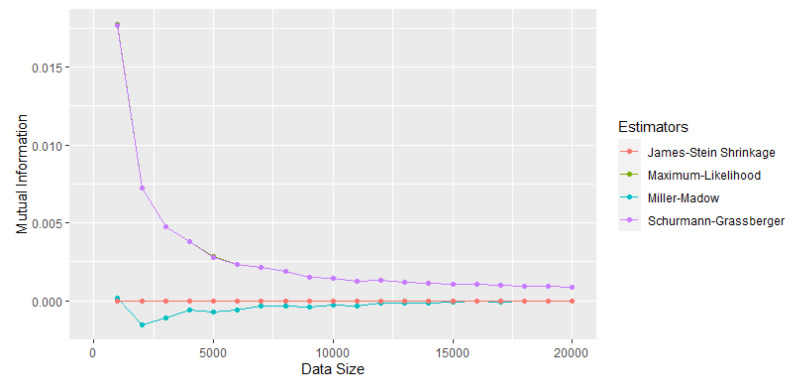
Comparison of mutual information estimators for a pair of independent tests [17].

**Figure 2:**
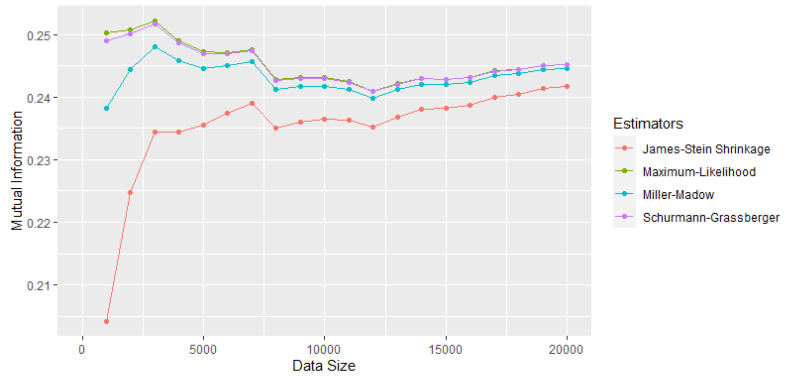
Comparison of mutual information estimators for a pair of correlated tests [17].

In [17], the ML, Miller–Madow, James–Stein, and Schurmann–Grassberger estimators were compared as the sample size increased. For the selection of the mutual information estimator, two pairs of variables were used, one of independent variables (see Figure 1) and the other of dependent variables (see Figure 2). It was concluded that, for more than 10,000 observations, the difference between the estimators is very small: 0.0074 for the pair of independent variables and 0.0065 for the dependent ones. However, the James–Stein estimator (shrinkage) was selected since the mutual information took the value of 0 for the independent variables, even for very small sample sizes.

Although for this particular case, only these mutual information estimators are being compared, a more comprehensive study of the entropy estimators can be found in [36]. The entropy estimator James–Stein shrinkage used to calculate mutual information in [17] is defined as

(8) $${\hat{H}}_{SH}=-\sum_{x\in K}{\hat{p}}_{x}^{SH}\log_{2}{\hat{p}}_{x}^{SH}$$

where

(9) $${\hat{p}}_{x}^{SH}=\hat{\lambda }t_{x}+\left(1-\hat{\lambda }\right){\hat{p}}_{x}^{ML}$$

with

(10) $$\hat{\lambda }=\frac{1-\sum_{x=1}^{k}{\left({\hat{p}}_{x}^{ML}\right)}^{2}}{\left(n-1\right)\sum_{x=1}^{k}{\left(t_{x}-{\hat{p}}_{x}^{ML}\right)}^{2}}$$

and

(11) $$t_{k}=\frac{1}{k}$$

### 2.4. MIRT-1 Method

As the distribution of the shrinkage estimator of the mutual information is unknown, a permutation test was performed to decide which values were significantly greater than 0. A permutation test is a statistical method used to determine the significance of a particular statistic by comparing it to a null distribution generated by randomly permuting the data. In this case, the permutation test determines whether the mutual information between two variables significantly differs from what would be expected by chance.

The null hypothesis ($H_{0}$) is that there is no relationship between the two variables, and any observed mutual information is due to chance. The alternative hypothesis ($H_{1}$) is that there is a significant relationship between the two variables, and the observed mutual information is not due to chance. If the *p*-value is less than a predetermined significance level, then the null hypothesis can be rejected in favor of the alternative hypothesis, indicating that there is a significant relationship between the two variables. The permutation test is applied following these steps:

1. Let $X=(x_{1},\;\ldots ,\;x_{n})$ and $Y=(y_{1},\;\ldots ,\;y_{n})$ be continuous random variables.
2. Construct the permuted samples $\left(X,\pi_{i}\left(Y\right)\right),\;\forall i=\bar{1,k}$ in such a way that the possible association between *X* and *Y* disappears, $\pi_{i}$ being the permutation *i* of elements of *Y*, i.e.,
  - $\pi_{i}\in S_{n},\;\forall i=\bar{1,k}$;
  - $\pi_{i}\neq \pi_{j}$, for i≠j;
  - $\pi_{0}$ is the identity of $S_{n}$.
3. Estimate the MI of the allowed samples to obtain ${\left\{Z_{i}\right\}}_{i=0}^{k}$, where $Z_{i}=MI\left(X,\pi_{i}\left(Y\right)\right)$.
4. The *p*-value associated with the test is calculated by
  (12) $$p\text{-value}=\frac{\sum_{j=1}^{k}I_{\geq Z_{0}}\left(Z_{i}\right)}{k},$$
  where $I_{\geq Z_{0}}\left(Z_{i}\right)$ is the indicator function defined by
  (13) $$1_{\geq Z_{0}}\left(x\right)=\left\{\begin{array}{ccc} 1 & if & x\geq Z_{0},\\ 0 & if & x<Z_{0}. \end{array}\right.$$
5. If p-value≥α, then the null hypothesis is not-rejected.

In [17], these steps were performed for each pair of random statistical tests, computing q=10,000 permutations in each case.

The MIRT-1 method proposed in [17] is described in the following steps:

1. Select PRNGs.
  – The selected generators must generate outputs that satisfy the randomness conditions.
2. Build the data samples using the selected generators.
  – Generate *n* sequences of random numbers of length *L* to be evaluated using the selected statistical randomness tests.
3. Evaluate each of the *n* sequences using the *k* statistical randomness tests to obtain the corresponding *p*-values for each $T_{i}$ test (with i=1,…,k).
4. Compute the MI between sequences of *p*-values to detect the possible presence of correlations.
  – Estimate the MI between pairs $\left(T_{i},T_{j}\right)$ of sequences of *p*-values to detect the presence of correlation.
  – In the case of the MIRT-1 method, the estimator used is the shrinkage estimator.
  – For a better interpretation in [17], the MI values were normalized, i.e., $MI^{\prime }\left(T_{i},T_{j}\right)=\frac{IM(T_{i},T_{j})}{H(T_{i})}$, where $H(T_{i})$ represents the entropy of the variable $T_{i}$.
5. Determine the significant correlations to conclude the correlation between the tests using the permutation test. The MI values are grouped in a triangular matrix
  $$M={\left(\begin{array}{cccc} H(T_{1}) & MI(T_{1},T_{2}) & \ldots & MI(T_{1},T_{k})\\ 0 & H(T_{2}) & \ldots & MI(T_{2},T_{k})\\ \vdots & \vdots & \ddots & \vdots \\ 0 & 0 & \ldots & H(T_{k}) \end{array}\right)}_{k\times k}$$
  where $MI(T_{i},T_{j})$ represents the MI between *i* and *j*. The diagonal of the matrix contains the values $H(T_{i})$ that represent the entropy of the variable $T_{i}$.

The complexity of the MIRT-1 method is estimated in [20] using the following:

(14) $$O(k^{2}\cdot q\left(n+d^{2}\right)),$$

where *k* represents the number of statistical tests to be analyzed, *q* is the number of permutations to determine the significance of the correlations, *n* is the length of the sequences of *p*-values analyzed, and *d* is the number of intervals in the discretization process. In practical applications, it is of interest to increase *k*, and the parameter *d* is selected in such a way as to increase the effectiveness of detection. Therefore, the feasible parameters to reduce are *q* and *n*, but the reduction in *n* can affect the effectiveness of the estimator, so, in this work, we will focus on reducing the value *q*.

## 3. Reducing the Complexity of the Method Based on Mutual Information to Analyze the Independence between the Statistical Tests of Randomness

This section presents the main contribution of this work: significantly reducing the complexity of the MIRT-1 method.

### 3.1. Solution Idea

To reduce complexity, it is proposed to eliminate the permutation test, which poses a new problem. In Step 5 of the MIRT-1 method, deciding which values of the mutual information are significant is necessary to conclude the correlation between the tests. Given the continuous random variables *X* and *Y*, decide if the value MI(X,Y) is significantly greater than 0, and thus conclude if there is some dependency between both variables. The hypothesis test asks the following:

(15) $$\left\{\begin{array}{cc} H_{0}: & MI(X,Y)=0\\ H_{1}: & MI(X,Y)>0 \end{array}\right.,$$

where $H_{0}$ is the null hypothesis that states the independence between *X* and *Y*, and $H_{1}$ is the alternative hypothesis, where there would be some association between *X* and *Y*.

To determine the significant values without using the permutation test, we propose to replace the James–Stein (shrinkage)-type estimator ${\hat{MI}}_{SH}$, whose distribution is unknown, with another estimator of mutual information whose distribution is known. The mean square error of the new estimator should be reasonably small for the selected sample size. In this paper, we will use a maximum-likelihood estimator transformation ${\hat{MI}}_{ML}$ whose distribution is known [51,52]. It is known that the maximum-likelihood estimator of entropy is normally distributed [54] asymptotically with mean

(16) $$E\left({\hat{H}}_{ML}-H\right)=-\frac{k-1}{2n}+\frac{1}{12n^{2}}\left(1-\sum_{i=1}^{k}\frac{1}{p_{i}}\right)+O\left(n^{-3}\right),$$

and variance,

(17) $$\sigma^{2}\left({\hat{H}}_{ML}\right)=\frac{1}{n}\left(\sum_{i=1}^{k}p_{i}\ln^{2}\left(p_{i}\right)-H^{2}\right)+\frac{k-1}{2n^{2}}+O\left(n^{-3}\right).$$

The mean can be calculated if the order $n^{2}$ is neglected. However, for the case of the variance, the terms of order *n* depend on the unknown probabilities. Therefore, the transformation of Equation (7) will be used since the mean and variance are known, and it is known that the normal distributes asymptotically when the number of degrees of freedom is greater than 30.

### 3.2. Selection of the Critical Value to Determine the Significant Correlations

To decide whether to reject the null hypothesis of independence between $\left(T_{i},T_{j}\right)$ in the new variant of the method, a priori knowledge of the distribution of the transformation $2n{\hat{MI}}_{ML}\left(T_{i},T_{j}\right)$ of estimator ${\hat{MI}}_{ML}$ will be used. The transformation $2n{\hat{MI}}_{ML}\left(T_{i},T_{j}\right)$ distributes $\chi^{2}$ (Equation (7)). For our case, I=J=d is the number of discretization intervals. Then,

(18) $$2n{\hat{MI}}_{ML}\left(T_{i},T_{j}\right)∼\chi_{{\left(d-1\right)}^{2}}^{2}.$$

To guarantee that the $\chi^{2}$ distribution of $2n{\hat{MI}}_{ML}\left(T_{i},T_{j}\right)$ approximates the normal distribution, the condition ${\left(d-1\right)}^{2}>30$ will be imposed on *d*, which implies that $d>1+\sqrt{30}=6.48$, concluding that, for values of d>6.48, we have

(19) $$2n{\hat{MI}}_{ML}\left(T_{i},T_{j}\right)∼N\left({\left(d-1\right)}^{2},\sqrt{2}\left(d-1\right)\right).$$

For the particular case of study, if we discretize d=10>6.48 intervals, we obtain ${\left(d-1\right)}^{2}={\left(9\right)}^{2}=81\gg 30$, which guarantees that the theoretical distribution of $2n{\hat{MI}}_{ML}\left(T_{i},T_{j}\right)$ closely approximates the normal distribution (Equation (19)). Using this theoretical distribution, the critical value corresponding to the α prefix can be taken, and therefore it is unnecessary to use the permutation test.

For the sampling distribution of $2n{\hat{MI}}_{ML}\left(T_{i},T_{j}\right)$ to be close to its theoretical normal distribution, it is convenient that the length *n* of the sequence of *p*-values is big enough. So,

(20) $$Z_{ML}=\frac{2n{\hat{MI}}_{ML}\left(T_{i},T_{j}\right)-{\left(d-1\right)}^{2}}{\sqrt{2}\left(d-1\right)}∼N\left(0,1\right)$$

distributes the normal asymptotically when ${\left(d-1\right)}^{2}\gg 30$. For an α significance level, the critical value for the right tail is $Z_{1-\alpha }$. Therefore, if the estimated values of $Z_{ML}$ are greater than the critical value, it can be concluded that there is a correlation between $T_{i}$ and $T_{j}$ with probability α that they are independent. For example, for α=0.01, if $Z_{ML}>Z_{1-0.01}=2.36$, it is concluded that the value $Z_{ML}$ is significant.

### 3.3. Method Modification Proposal

From Step 1 to Step 3, the MIRT-1 method remains unchanged. In Step 4, in [17], the shrinkage estimator ${\hat{MI}}_{SH}$ was used, while now it is proposed to use the ML estimator ${\hat{IM}}_{ML}$. In Step 5, in [17], the values of ${\hat{MI}}_{SH}$ were normalized. A permutation test was applied; on the other hand, in this new method used to determine the significant values of ${\hat{MI}}_{ML}$, the transformation $Z_{ML}$ is computed, and knowledge of the normal distribution of $Z_{ML}$ will be used.

The application of the new MIRT-2 method from this modification is as follows (the steps that changed concerning MIRT-1 are indicated in bold):

1. Select PRNGs.
  – The selected generators must generate outputs that satisfy the randomness conditions.
2. Build the data samples using the selected generators.
  – Generate *n* sequences of random numbers of length *L* to be evaluated using the selected statistical tests of randomness.
3. Evaluate each of the *n* sequences using the *k* statistical tests of randomness to obtain the corresponding *p*-values for each $T_{i}$ test (with i=1,…,k).
4. Compute the MI between sequences of *p*-values to detect the presence of correlations.
  – Calculate the MI between pairs $\left(T_{i},T_{j}\right)$ of sequences of *p*-values to detect the presence of correlation.
  – The MI estimator used is the ML ${\hat{MI}}_{ML}$
5. Calculate $Z_{ML}$ and compare it with the critical value associated with the default α value. If ${\hat{Z}}_{ML}>Z_{1-\alpha }$, the null hypothesis of independence between the tests of randomness is rejected.

Table 2 summarizes the parameters chosen for the MIRT-1 and MIRT-2 methods.

The complexity of the MIRT-1 method was calculated in [20]

(21) $$O(k^{2}\cdot q\left(n+d^{2}\right)).$$

The modification proposed in this paper reduces it to

(22) $$O(k^{2}\left(n+d^{2}\right)),$$

since not applying the permutations test is equivalent to taking q=1. The reduction achieved is of the order

(23) $$\frac{k^{2}\cdot q\left(n+d^{2}\right)}{k^{2}\left(n+d^{2}\right)}=q,$$

so the modified method is expected to be about *q*-times faster. For example, in [17,20], q=10,000 was used; for this case, the MIRT-2 method would have a complexity 10,000 times lower than MIRT-1.

The replacement of the shrinkage estimator ${\hat{MI}}_{SH}$ of mutual information used in the MIRT-1 method with the maximum-likelihood estimator ${\hat{MI}}_{ML}$ allows complexity to be reduced since the permutation test is eliminated and the known distribution of the new estimator is used to set the critical value and select the significant values. This modification could affect the ability to detect correlations if appropriate measures are not taken. To avoid this impact, it is proposed to apply the MIRT-2 method with a sample size equal to 10000, and it is recommended not to reduce this value while this ${\hat{MI}}_{ML}$ estimator is used. This recommendation is based on the results of Figure 2 of [17] since, for this sample size, the difference between the values of the estimators is very small and the ability to detect correlations is not affected.

## 4. Experimental Validation

In this section, the reduction in the complexity of MIRT-2 to MIRT-1 will be verified experimentally.

### 4.1. Experimental Check of Normality of $Z_{ML}$

#### 4.1.1. Design of Experiment 1

For random sequences, the *p*-values generated by statistical randomness tests follow a uniform distribution with values between 0 and 1 [9]. For this reason, the experiments generated data that followed the same distribution. To study the normality of the transformation $Z_{ML}$ and the pairs $(X_{i},Y_{i})\;,\;i=1,\;\ldots ,\;1000$ of independent and identically distributed random variables, where X,Y∼U(0,1), $X=\{x_{1},x_{2},...,x_{10,000}\}$ and $Y=\{y_{1},y_{2},...,y_{10,000}\}$. Subsequently, the MI between the pairs was calculated using the ML estimator, and 1000 ${\hat{MI}}_{ML}$ observations were obtained. The MI values were transformed using Equation (20) to obtain a sample of 1000 $Z_{ML}$ values. The Anderson–Darling and Kolmogorov–Smirnov tests were applied to verify the normality of $Z_{ML}$.

#### 4.1.2. Results

Table 3 shows *p*-values for the Anderson–Darling and Kolmogorov–Smirnov normality tests.

**Figure 3:**
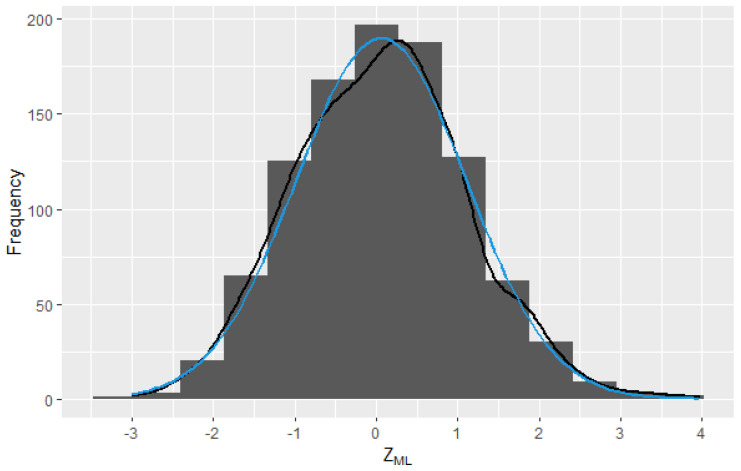
Sampling distribution of the 1000 observations of $Z_{ML}$ obtained from pairs $\left(X_{i},Y_{i}\right)$.

For all cases, the p-value>0.05; therefore, there is insufficient evidence to reject the null hypothesis of normality. In Figure 3, a histogram with the values of $Z_{ML}$ and the estimate of the kernel density (KDE) of the sample data (black) and the curve of normal density (blue) can be observed. The blue line represents the theoretical normal probability density function, while the black curve represents a smoothed density estimate using a kernel density estimation technique.

**Figure 4:**
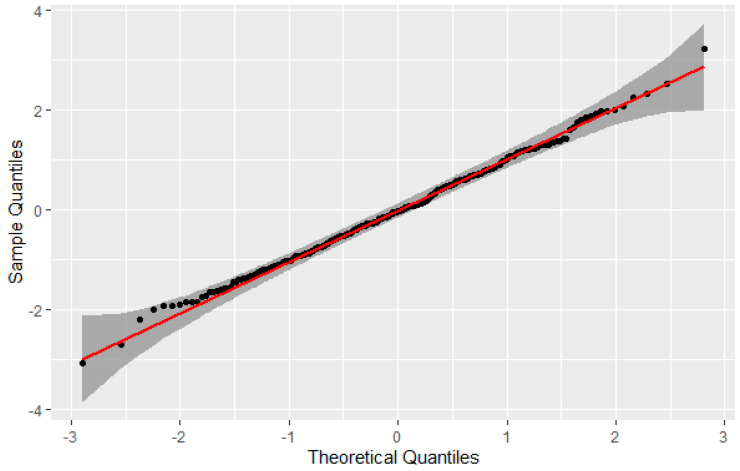
Quantile plot of the 1000 observations of $Z_{ML}$ obtained from pairs $\left(X_{i},Y_{i}\right)$.

In the Q-Q graph of Figure 4, it can be seen that the observed values are close to the expected ones. Through normality tests, it was possible to verify experimentally that the transformation $Z_{ML}$ follows a normal distribution under the theoretical argumentation presented in Section 3.2. The red line in the Q-Q plot graph represents the theoretical quantiles of a standard normal distribution, and it serves as a reference line for comparing the distribution of the plotted data to the normal distribution.

### 4.2. Analysis of the Effectiveness of the Proposed Variant

#### 4.2.1. Design of Experiment 2

Based on the idea of the previous section, the MI between the NIST statistical randomness tests was calculated using the MIRT-2 method. For this, the ML estimator was used, and the symmetric matrix of dimension 17×17 was obtained with the values of the MI. It is important to highlight that for selecting the *p*-values, the approach proposed by [20] was followed. All sequences that did not comply with the requirement were discarded. There was a required number of cycles for the random excursions and random excursions variant tests, thus generating *p*-values equal to 0. Under the standard normal distribution and the independence assumption, a significance level α=0.001 corresponds to a critical value CV=3.090232. Therefore, if $Z_{ML}>3.09$, the null hypothesis of independence is rejected. Following Step 5 of the MIRT-2 method, those greater than the critical value were selected as significant values of the $Z_{ML}$.

#### 4.2.2. Results

**Figure 5:**
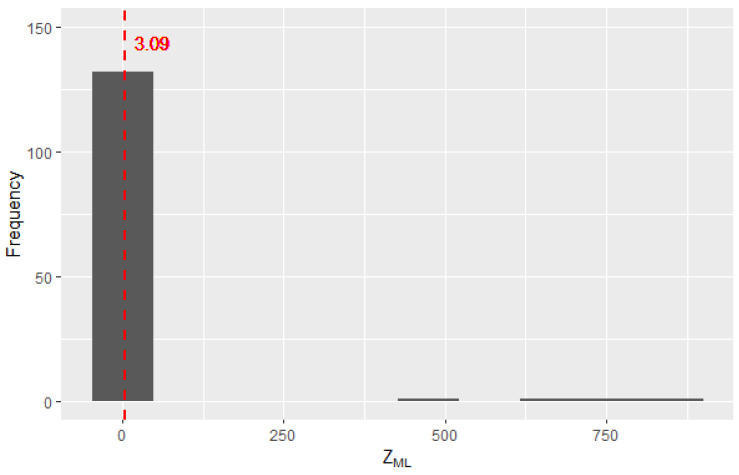
Distribution of the 136 observations of $Z_{ML}$ for values between 0 and 1000.

**Figure 6:**
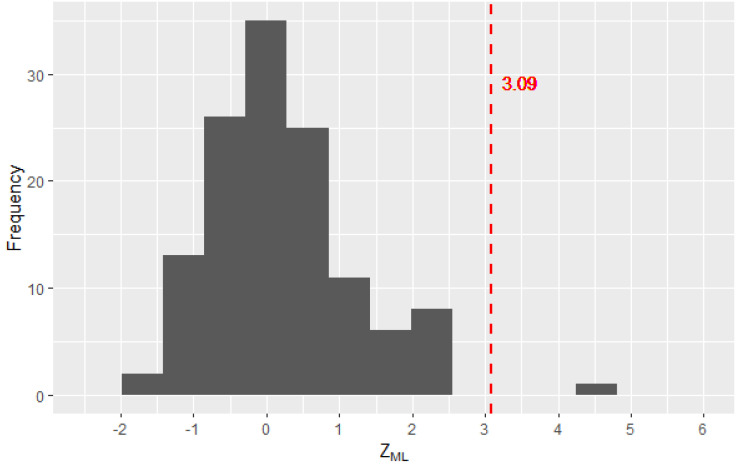
Distribution of $Z_{ML}$ observations for values between 0 and 6.

In Figure 5, the histogram is represented with the k(k−1)/2=136 observations of $Z_{ML}$ for the values of the mutual information between the pairs of statistical tests of randomness. High values of $Z_{ML}$ (greater than 300) are seen on the right, indicating possible correlations between test pairs. In Figure 5 and Figure 6, the dotted line represents the critical value selected for the MI.

In Figure 6, only the observations of $Z_{ML}$ are represented for the values between 0 and 6. It can be noted that the values of $Z_{ML}$ smaller than the critical value behave following a normal distribution, as expected under the independence hypothesis.

**Figure 7:**
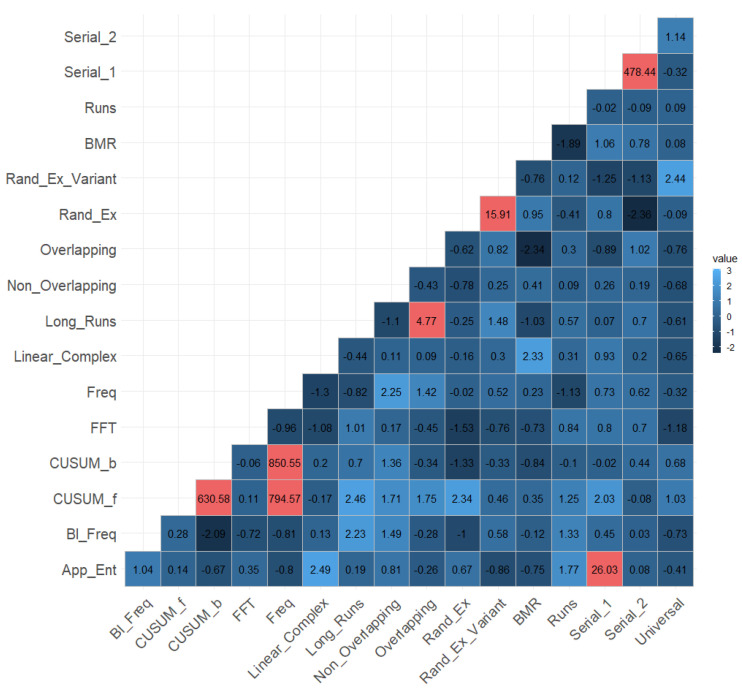
Mutual information matrix with the values of $Z_{ML}$ between the pairs of statistical tests for randomness.

Figure 7 shows the mutual information matrix with the significant values for the selected critical value. The values of $Z_{ML}$ greater than CV=3.09 are indicated in red.

Table 4 compares the correlations detected by the two variants. The paper presented by [17] did not consider the pre-processing of invalid sequences for the random excursions and random excursions variant tests. For this reason, the correlations detected with the MIRT-1 method in [20] and the new MIRT-2 variant proposed in this article will be compared.

The correlation between the CUSUM (b) and non-overlapping template tests was not detected by the new MIRT-2 method, indicating that the new variant for this case loses efficacy concerning MIRT-1. This may be due to substituting the shrinkage estimator for the ML. However, according to what was expressed in [20], this correlation is quite small, so its detection may be due to an error or not easy to detect.

#### 4.2.3. Design of Experiment 3

To verify the reduction in complexity of the new variant of the method concerning MIRT-1, the experiments presented in [17] were repeated. The methods were implemented in *R* using the entropy library in a computer running Windows 10 (64-bit) operating system, Intel Core i7-3770 3.40 GHz CPU, and 32 GB RAM. The MI with the two versions, MIRT-1 and MIRT-2, was calculated, and the times were compared. This experiment was repeated ten times. Results are shown in Table 5.

As seen in Table 5, the times were reduced on average 19,444-fold approximately, where 19,444>q=10,000. It can be seen that the reduction in time observed in practice is even greater than the expected theoretical reduction.

**Figure 8:**
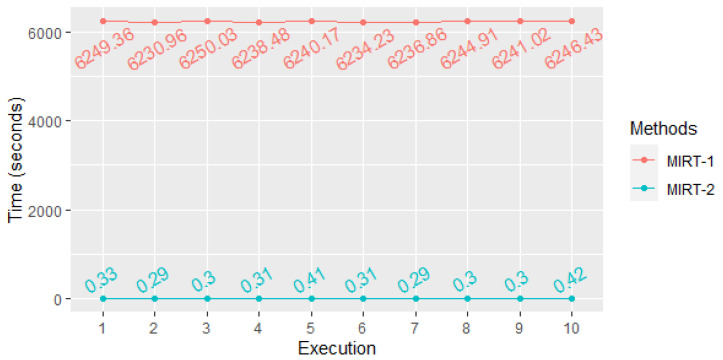
Representation of the execution time of the methods for ten experiments.

The times for detection of correlations between the 17 tests of the NIST battery with sequences of *p*-values of length 10,000 decreased from approximately 1.7 h to less than 1 s (see Figure 8).

### 4.3. Analysis of the Consistency and Stability of the MIRT-2 Method

In this section, a detailed analysis of the consistency of the method MIRT-2 and MIRT-1 and the stability of MIRT-2 will be carried out.

#### 4.3.1. Consistency

To analyze the consistency of the estimators ${\hat{MI}}_{ML}$ and ${\hat{MI}}_{SH}$ of mutual information, a Bland–Altman test was performed. The Bland–Altman test is a statistical method used to assess the agreement between two measurement methods. In this case, the experiment aimed to check the consistency between the estimators ${\hat{MI}}_{ML}$ and ${\hat{MI}}_{SH}$.

**Figure 9:**
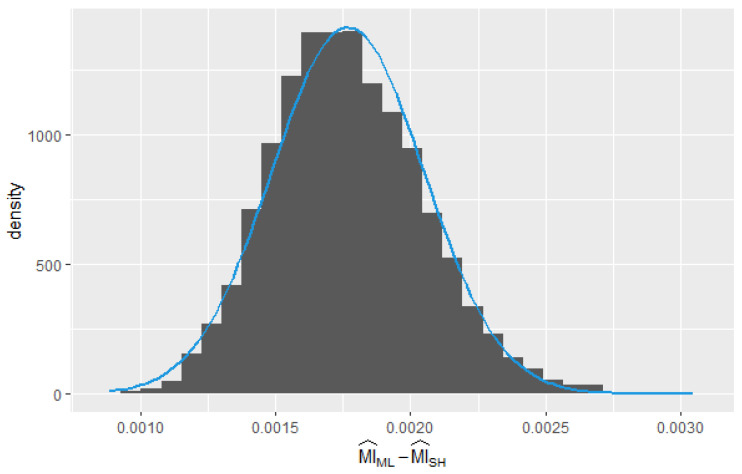
Distribution of the differences in the estimators ${\hat{MI}}_{ML}$ and ${\hat{MI}}_{SH}$.

To perform the Bland–Altman test, the following steps were taken:

1. Generate 10,000 samples of independent and identically distributed random variables U(0,1).
2. Calculate the mutual information using both ${\hat{MI}}_{ML}$ and ${\hat{MI}}_{SH}$ estimators for each sample.
3. Calculate the mean and difference between the two methods for each sample. To check the assumptions of normality of the differences, a test for normal distribution, such as the Shapiro–Wilk or Kolmogorov–Smirnov test, can be conducted for the hypothesis that the distribution of the observations in the sample is normal (Figure 9)) (if p<0.05, then reject normality).
4. Calculate the mean difference and the LOAs (limits of agreement) (mean difference ±1.96 times the standard deviation of the differences.)
5. Interpret the results. If the mean difference is close to zero and the limits of agreement are narrow, then the two methods are considered to be in good agreement.

If the mean difference is close to zero and the LOA is narrow, it suggests that the two methods are consistent and can be used interchangeably. On the other hand, if the mean difference is far from zero and/or the LOA is wide, it suggests that the two methods are inconsistent and cannot be used interchangeably.

In this case, the test was performed on 10,000 comparisons of the two estimators. The main measures are summarized in Table 6, resulting in a bias of 0.001768216 and a standard deviation of bias of 0.0002812867. The upper and lower LOAs were calculated to be 0.002319538 and 0.001216894, respectively.

**Figure 10:**
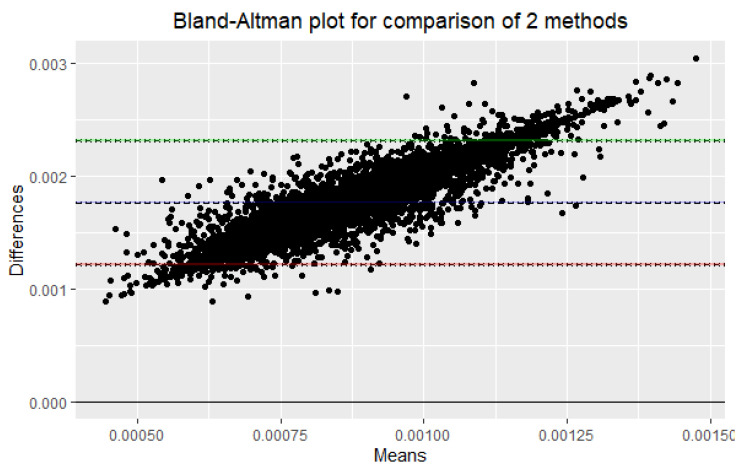
Bland–Altman plot for comparison of the estimators ${\hat{MI}}_{ML}$ and ${\hat{MI}}_{SH}$.

The bias is within the LOA, indicating that there is no significant difference between the two measures. However, the bias is not zero, indicating that there is some systematic difference between the two measures. The upper and lower LOAs are relatively narrow (Figure 10), indicating good agreement between the two measures.

Overall, the results of the Bland–Altman test suggest that the two measures being compared are in good agreement, with a small systematic difference between them.

#### 4.3.2. Stability

To assess the stability of this estimator, a stability test was carried out using a bootstrap test with 10,000 repetitions.

The hypothesis being tested is that the ${\hat{MI}}_{ML}$ estimator is stable, producing consistent results when applied to different data samples. The null hypothesis is that the ${\hat{MI}}_{ML}$ estimator is unstable, producing inconsistent results when applied to different data samples.

To perform the bootstrap test, multiple data samples were randomly selected with replacements from the original dataset. The ${\hat{MI}}_{ML}$ estimator was applied to each sample, and the resulting mutual information values were recorded. This process was repeated 10,000 times to generate a distribution of mutual information values.

**Figure 11:**
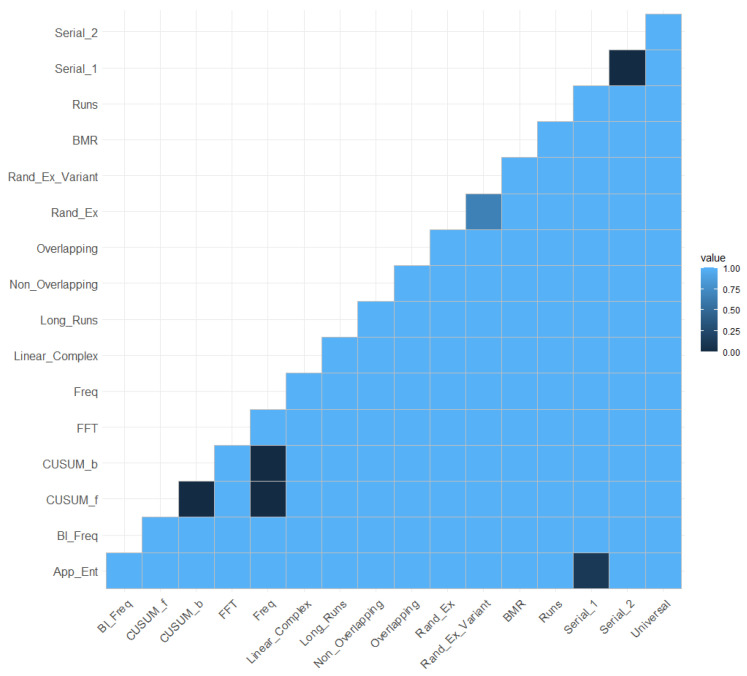
Matrix to illustrate the stability of the MI estimator ${\hat{MI}}_{ML}$.

This was carried out for each dataset of each of the NIST statistical tests. The results showed that of the seven correlations detected with the original data, after carrying out the bootstrap test, six correlations were maintained (Figure 11). It can be concluded that, in general, the MIRT-2 method presents good stability. It would be necessary to study in depth the reason why the correlation between the overlapping and longest run tests did not remain stable.

## 5. Conclusions

In this work, we reduced the complexity of the MIRT-1 method proposed by [17] *q*-fold by modifying the selection criteria for significant values. The complexity reduction was estimated theoretically and confirmed through experimentation. In addition, it was concluded that this modification does not significantly affect the method’s effectiveness in detecting correlations. Therefore, it was proposed for this modification to be implemented in future applications of the MIRT-1 method to enhance its efficiency. As directions for future work, it is recommended to apply the MIRT-2 method to analyze the correlation in other batteries of statistical tests, such as the batteries analyzed in [18,19], with the MIRT-1 method. Some of the batteries that can be studied are ENT, FIPS 140-2, DieHarder, TufTests, and TestU01. On the other hand, it is proposed to continue reducing the complexity of the MIRT-2 method by reducing the value of *n*. Although it is important to note that reducing *n* may increase the mean square error of the ${\hat{MI}}_{ML}$ estimator used in MIRT-2, this causes a decrease in the method’s effectiveness. The challenge is to reduce n and complexity without losing effectiveness.

## Figures and Tables

**Table 1:** Rules for selecting the number *k* of intervals for the discretization.

Rules	*k*
**Sturges** [39]	$1+\log_{2}\left(n\right)$
**Cochran** [40]	$\left\lfloor \sqrt{n/5}\right\rfloor$
**Rice** [41]	$\left\lfloor 2\sqrt[{3}]{n}\right\rfloor$
**Cencov** [42]	$\left\lfloor \sqrt[{3}]{n}\right\rfloor$
**Bendat–Piersol** [43]	$\left\lfloor 1.87{\left(n-1\right)}^{0.4}\right\rfloor$
**Larson** [44]	$1+\lfloor 2.2\log_{2}\left(n\right)\rfloor$
**Velleman** [45]	$\left\lfloor 2\sqrt{n}\right\rfloor$ if n≤100
	$\left\lfloor 10\log_{10}\left(n\right)\right\rfloor$ if n>100
**Doane** [46]	$1+\log_{2}\left(n\right)+\log_{2}\left(1+\frac{\sqrt{b_{1}}}{\sigma \sqrt{b_{1}}}\right)$
**Mosteller–Tukey** [47]	$\left\lfloor \sqrt{n}\right\rfloor$
**Terrell–Scott** [48]	$\sqrt[{3}]{2n}$
**Ishikawa** [49]	6+⌊n/50⌋

**Table 2:** Parameters used for the MIRT-1 and MIRT-2 methods.

Method	MIRT-1	MIRT-2
Distribution of the *p*-values	U(0,1)	U(0,1)
MI estimator	shrinkage (SH)	maximum-likelihood (ML)
Discretization	k = 10	k = 10
Selection of significant values	Comparison with the critical value obtained by the permutation test.	Comparison with the critical value of the normal distribution for the α prefix.

**Table 3:** Tests for normality for the values of $Z_{ML}$.

Normality Test		*p*-Value
Anderson–Darling	A=0.47518	0.2395
Kolmogorov–Smirnov	D=0.019679	0.8334

**Table 4:** Comparison of the correlations detected by the MIRT-1 and MIRT-2 methods.

	MIRT-1	MIRT-2
App. Ent.	≈Serial 1	≈Serial 1
CUSUM (f)	≈CUSUM (b) ≈Frequency	≈CUSUM (b) ≈Frequency
CUSUM (b)	≈Frequency ≈Non-Overlapping	≈Frequency
Long. Run	≈Overlapping	≈Overlapping
Random Ex.	≈Random Ex. Variant	≈Random Ex. Variant
Serial 1	≈Serial 2	≈Serial 2

**Table 5:** Execution time in seconds of the methods for ten experiments.

Execution	MIRT-1	MIRT-2	Quotient
1	6249.36	0.33	18,937.45
2	6230.96	0.29	21,486.07
3	6250.03	0.3	20,833.43
4	6238.48	0.31	20,124.13
5	6240.17	0.41	15,219.93
6	6234.23	0.31	20,110.42
7	6236.86	0.29	21,506.41
8	6244.91	0.30	20,816.37
9	6241.02	0.30	20,803.40
10	6246.43	0.42	14,872.45
μ	6241.245	0.326	19,144.92
σ	40.713	0.002	17,415.26

**Table 6:** Bland–Altman test measurements.

Bias	0.001768216
The standard deviation of bias	0.0002812867
Upper LOA	0.002319538
Lower LOA	0.001216894
Mean of differences/means	202.4483
